# *In Situ* Grazing Incidence X‑ray Total Scattering Reveals the Effect of the “Two-Step” Method for the Anodization of Aluminum Surfaces

**DOI:** 10.1021/acsami.5c05251

**Published:** 2025-08-11

**Authors:** Nicolas P. L. Magnard, Giuseppe Abbondanza, Laura S. Junkers, Lorena Glatthaar, Andrea Grespi, Alexander Spriewald Luciano, Fernando Igoa Saldaña, Ann-Christin Dippel, Nikolay Vinogradov, Herbert Over, Kirsten M. Ø. Jensen, Edvin Lundgren

**Affiliations:** † Department of Chemistry, University of Copenhagen, DK-2100 Copenhagen, Denmark; ‡ Department of Physics, 11248 Chalmers University of Technology, Chalmersplatsen 4, Gothenburg 41296, Sweden; § Institute of Physical Chemistry, 4321 Justus Liebig University, Heinrich-Buff-Ring 17, D-35392 Giessen, Germany; ∥ Division of Synchrotron Radiation Research, 5193 Lund University, Professorsgatan 1, 22363 Lund, Sweden; ⊥ NanoLund, Lund University, Professorsgatan 1, 22363 Lund, Sweden; # 28332 Deutsches Elektronen-Synchrotron DESY, Notkestraße 85, 22607 Hamburg, Germany; ∇ MAX IV Laboratory, Lund University, Fotongatan 2, 22484 Lund, Sweden

**Keywords:** X-ray total scattering, grazing incidence, *in situ* studies, NP-AAO, amorphous oxide

## Abstract

Thanks to its ability to form a lattice of self-ordered nanosized pores, nanoporous anodic aluminum oxide (NP-AAO) is a promising material with diverse applications, for example, as membrane for the controlled deposition of catalysts. NP-AAO is obtained by the anodization of an aluminum substrate under specific pH and voltage conditions. However, the growth mechanism of the oxide is still under debate. We shed light on this process by investigating structural changes at the atomic scale using pair distribution function (PDF) analysis. We thus performed *in situ* X-ray total scattering experiments under grazing incidence conditions during the anodization of aluminum substrates exhibiting different crystallographic facets. By doing so, we were able to track the evolution of the local structure of aluminum oxide species forming at the oxide-electrolyte interface over time.

## Graphical abstract

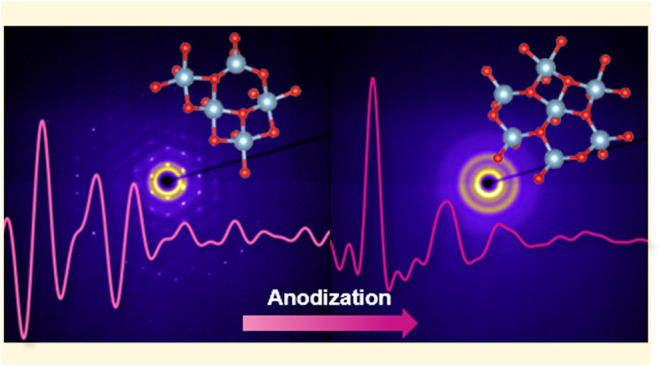

## 1 Introduction

Over the past few decades, nanoporous anodic aluminum oxide (NP-AAO) has gained significant attention due to its unique properties. These include, among others, its self-ordered hexagonal nanoporous structure, the ability to easily control pore size as well as interpore distance through the anodization conditions, its low cost, and thermal stability up to 800 °C.^1–7^ As a result, NP-AAO is now widely used as a template for the nonlithographic synthesis of various nanomaterials, such as nanodots,^8–10^ nanowires,^11–15^ and nanotubes.^16,17^

NP-AAO can be easily fabricated by anodizing aluminum in a number of acidic electrolytes, including sulfuric acid,^18–20^ oxalic acid,^21–23^ phosphoric acid,^3,24^ and chromic acid,^25^ but also in alkaline electrolytes.^26^ On the other hand, barrier-type alumina thin films can be formed in neutral electrolytes with a pH range of 5–7.^4^ In this study, we focus exclusively on porous-type anodic alumina. The pore size and interpore distance of NP-AAO can be adjusted from a few to hundreds of nanometers by varying the anodization potential.^3,27,28^ A thin oxide barrier layer exists between the porous NP-AAO layer and the aluminum substrate. It has been established over the years that the thickness of this barrier layer, as well as the pore size and interpore distance, have linear relationships with the anodization potential.^22,27,29–31^ Moreover, it has been shown that during a first anodization of aluminum, the resulting NP-AAO exhibits a structure with two distinct regions: an upper layer formed at the initial stages of the anodization process, typically more disordered, and a lower layer with increasingly ordered pores as anodization progresses.^32^ By subsequently removing this oxide layer, often via selective etching, one exposes a nanoconcave pattern on the underlying aluminum surface. These concaves serve as nucleation sites in a second anodization step, enabling the formation of NP-AAO with significantly improved pore uniformity and long-range ordering in the upper layer of the formed AAO. This approach, known as the "two-step anodization"method, was developed specifically to produce well-ordered porous alumina structures beyond what is achievable with a single anodization process.^32^ In this method, the oxide is selectively removed by wet etching in a chromic/phosphoric acid solution, creating a substrate patterned with nanoconcaves that guide the subsequent growth of the nanoporous structure.

The mechanism of pore growth in NP-AAO has been the subject of continuous investigation for decades and is still a topic of debate.^2,25,33–35^ Previous models can be broadly categorized into two types: those that consider the electric field as the driving force for pore growth and self-organization and those that consider mechanical stress as the driving force. Some theories propose that electric field-assisted dissolution is responsible for pore formation and growth in NP-AAO.^27^ These models suggest that the thickness of the oxide barrier layer is the result of a competition between oxidation and dissolution reactions at the bottom of the pore, with the high electric field aiding in the dissolution of the oxide, resulting in a faster dissolution rate than that during open-circuit chemical dissolution. On the other hand, models based on mechanical stress suggest that a significant volume expansion occurring at the oxide/metal interface during the oxidation reaction provokes the formation of cracks in the oxide layer which, in turn, expose metal to the electrolyte and promote the dissolution of metal ions and their subsequent incorporation into new oxide. This volume expansion, known as the Pilling-Bedworth (PB) ratio, can be higher than 2.0 under certain conditions (PB ratios above 1 lead to uniform and crack-free aluminum barrier oxides).^36,37^ However, the confined volume expansion at the metal/oxide interface during anodization can generate compressive stress in the growing oxide layer. This stress is partially relieved by the outward migration of Al^3+^ ions and the associated formation of vacancies in the oxide lattice, which promote local plasticity and structural relaxation.^38^

Most models describing pore growth are based on *ex situ* observations as there has been a lack of *in situ* experimental investigations to probe structural evolution on the atomic scale. While X-ray diffraction can be used for structure analysis of crystalline electrode materials during electrochemical reactions, it is not suitable for studying the atomic arrangement of NP-AAO because of its amorphous nature.^2^ The structure of disordered systems can, however, be studied using direct-space investigation of the total scattering signal (including both Bragg and diffuse scattering) via pair distribution function (PDF) analysis.^39,40^ This technique makes it possible to extract local structural motifs of systems lacking long-range order, such as bulk NP-AAO.^41^

Another experimental technique that is capable of providing structural insights into the atomic structure of amorphous materials is the analysis of X-ray absorption extended fine structure (EXAFS) data. However, low photon energy necessary for probing of the low-Z absorption edges (Mn, Cr, Al, etc.) results in a small probing depth, making EXAFS measurements on low-Z elements inapplicable for the study of Al surfaces. In fact, we have recently shown that, based on the geometry and the optical path length of X-rays through our electrochemical flow-cell for X-ray measurements, the EXAFS approach is only suitable for materials with absorption edges above 8 keV. Since elements such as Mn, Cr, and Al have absorption edges below this limit, they are undetectable with this technique.^42^ Therefore, in order to investigate the evolution of the local NP-AAO structure over time, we performed *in situ* grazing incidence total scattering (TS) measurements during the anodization of Al single crystals, i.e., the formation of the NP-AAO.

In the context of oxide growth on crystalline substrates, understanding the structural evolution is crucial, regardless of whether the resulting oxide is crystalline or amorphous. Recent work on crystalline aluminum oxide^43^ has provided key insights into the interface interactions and stability of oxide layers. Although the current study focuses on the growth of an amorphous porous oxide, the significance of exploring these structural transformations shares similar importance, as both approaches aim to unravel the mechanisms that govern oxide formation and stability, with implications for catalytic, electronic, and protective applications.

We discuss various aspects of our work, including the data analysis strategy used to exclude the Bragg reflections from the signal to obtain a clear PDF, the effect of two experimental grazing incidence geometries, i.e., slightly above and below the critical angle, and the impact of different crystal orientations on the evolution of the Al–O interatomic distance. We also discuss the effect of the so-called “two-step” method on the growth process. In order to achieve this, we compare the structural evolution of the aluminum oxide network in NP-AAO formed from pristine Al surfaces versus pre-etched substrates, assessing how the pre-patterning step impacts the final overall structural order.

## 2 Materials and Methods

### 2.1 Anodization of Aluminum

In this study, we focus on the anodization behavior of Al(111) over both anodization steps of the two-step method: Al(110) for the second anodization step and Al(100) for the first step. This choice was based on the availability and completeness of the data for each substrate. For Al(111), we obtained consistent and reproducible results across both steps, allowing for a more comprehensive analysis. In the case of Al(110), only the second anodization step yielded reliable data, and for Al(100), only the first step was successfully analyzed. The data sets for the incomplete steps, while informative, were not robust enough for a full comparison but are still included for reference where applicable. These differences are acknowledged as limitations of the experimental data and should be taken into account in interpreting the results. Nevertheless, including these incomplete data sets allows for a comparative analysis across the substrates, highlighting both the strengths and the limitations of the collected data. The crystals had purity 6 N, surface normal aligned with an accuracy of ≈0.1°, and top surface polished to an average roughness *R*_a_ < 0.03 μm (Surface Preparation Laboratory, The Netherlands). Upon air exposure, a native amorphous Al_2_O_3_ layer a few nanometers thick forms spontaneously on the surface.^2^ The crystals had a top-hat shape with an outer diameter of 13 mm, an inner diameter of 6 mm, and a total height of 6 mm. A schematic of the experimental setup is shown in Figure 1. The Al specimens acted as the working electrode in a two-electrode electrochemical flow-cell made of polyether ether ketone (PEEK), which was designed for X-ray measurements and used in numerous previous *in situ* experiments combining electrochemistry with small- and wide-angle X-ray scattering.^13,44,45^ A Pt rod was used as a counter electrode, and a thermocouple, inserted in a PEEK sleeve, was used to monitor the temperature during the experiment. A peristaltic pump (Cole-Parmer Masterflex) was used to circulate the anodizing electrolyte through PTFE tubing, to and from the flow-cell, while a Kikusui PBZ-20-20 programmable bipolar power supply was used to drive the anodization. During the reaction, the electrochemical current was recorded with a digital multimeter connected in series to the power supply.

**Figure 1:**
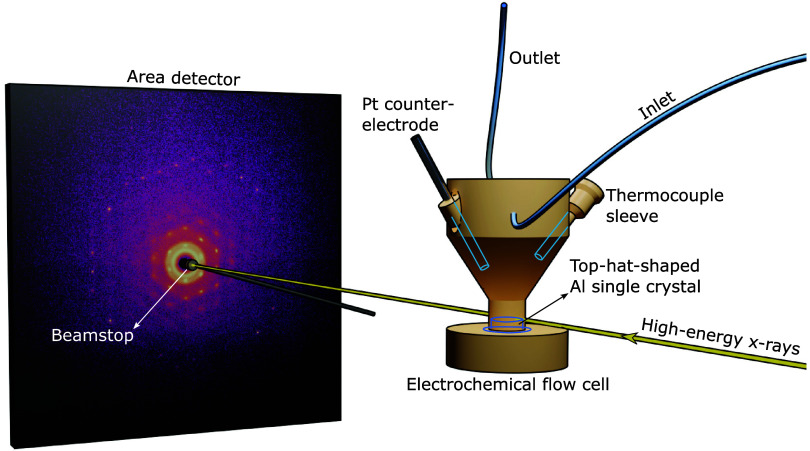
Schematic representation of the experimental setup.

Prior to the experiment, the flow-cell, the tubing, and the Pt rod were cleaned by flowing 20% HNO_3_ for 3 h, followed by a rinsing flow of 2 L ultrapure H_2_O (resistivity = 18.2 MΩ cm). The glassware was cleaned by soaking in 20% H_2_SO_4_:HNO_3_ 1:1 solution for 3 h and then rinsed thoroughly in ultrapure H_2_O. Ultrapure H_2_O and reagent-grade chemicals were used to prepare the electrolyte solution employed in this work, i.e., 0.3 M H_2_SO_4_ (sulfuric acid), and the anodizing potential was 25 V. Such conditions of electrolyte composition and potential are well known to lead to the formation of NP-AAO.^3,5,6^ The duration of the anodization was 2.5 h, and the temperature was kept constant at 14 ± 1 °C by placing the electrolyte bottle in a cold bath of water and ethylene glycol.

For the synthesis of NP-AAO, the “two-step” anodization method was used, which is known to lead to highly ordered nanoporous domains.^32^ Briefly, it consists of a first anodization, which leads to the formation of a disordered porous structure, followed by wet chemical stripping of the oxide and a second anodization step under the same anodization conditions. The oxide removal in our experiment was performed by soaking the specimens in a selective etching solution, i.e., a mixture of 0.185 M H_2_CrO_4_ and 0.5 M H_3_PO_4_, at 60 °C for 3 h. The oxide stripping results in a substrate patterned by nanoconcaves that guide the growth of the nanoporous honeycomb structure in the second anodization leading to well-ordered nanopores.

### 2.2 *In Situ* X-ray Total Scattering Measurements

Time-resolved X-ray TS experiments were performed at the P21.1 beamline at the storage ring PETRA III (DESY, Germany).^46^ Using compound refractive lenses (CRLs), an X-ray beam with an energy of 101.5 keV was focused to 2 × 150 μm^2^ (vertical by horizontal, full width at half-maximum). Each sample was aligned in height and tilt angles so that the Al surface was parallel to the incident X-ray beam and, furthermore, at half height of the vertical beam size to position the beam footprint at the center of rotation of the goniometer when the surface was set to the incidence angle. A PerkinElmer XRD1621 amorphous Si flat panel detector was mounted at a distance of 400 mm from the center of rotation of the goniometer with the primary beam hitting roughly the center of the detector area, yielding an instrumental *Q*_max_ of 23.75 Å^–1^. Detector images were acquired continuously during the anodization with a time resolution of 1 s. The X-ray beam intensity remained constant within 1% throughout the experiments. The calibration of the measurement geometry was done by collecting the diffraction pattern of a powder of the Ni standard inside a kapton capillary.

Additionally, another time-resolved X-ray TS experiment was carried out at the ID31 beamline at the ESRF (Grenoble, France). An X-ray beam with an energy of 70.0 keV was focused to 5 × 30 μm^2^ (vertical by horizontal, full width at half-maximum). The alignment procedure was performed in the same way as described above. A Dectris Pilatus3 X CdTe 2 M 2D detector was put at a distance of 282 mm from the center of rotation of the goniometer to collect the TS signal, with the direct beam hitting roughly the center of the detector. This geometry allowed for an instrumental *Q*_max_ of 14.60 Å^–1^. For this experimental setup, detector images were acquired continuously with a time resolution of 3 s until no further change in the TS pattern was observed. The calibration of the measurement geometry was done by collecting the diffraction pattern of a powder of the CeO_2_ standard inside a Kapton capillary.

### 2.3 Data Processing

The analysis of the PDFs posed several technical challenges, with the main one being that the amorphous oxide grows on top of a monocrystalline substrate. The resulting very intense Bragg reflections dominate the total scattering signal, especially in the early stage of the process. The fact that the NP-AAO signal is a weak diffuse scattering overlaying these Bragg peaks makes the corresponding data analysis significantly more difficult than that for conventional PDF experiments. Moreover, diffuse scattering in grazing incidence condition is challenging to measure as it has a weak scattering intensity.^47,48^

First, the raw images were corrected using the detector gain map because the gain of each individual pixel varies from one another. Each detector image was thus multiplied by an array containing the gain value for each individual detector pixel. The calibration of the detector images was performed using the pyFAI package^49^ of Dioptas.^50^ Each detector image was then integrated with the same software. The scattering data from a cell containing only electrolyte was used for background subtraction. An example of this subtraction is shown in Figure S1. The corrected TS data were then reduced and PDFs were obtained using the pdfgetx3 package.^51^ It should be noted, however, that the Ni and CeO_2_ powders contained in a glass capillary were used for geometry calibration in pyFAI. Due to the significantly different geometry compared to the real sample, i.e., Al single crystals, these measurements were not suitable for deriving instrumental parameters such as *Q*_damp_ and *Q*_broad_. Accordingly, no further refinement of a structural model onto the experimental PDF was carried out in this study.

### 2.4 Strategy for the Treatment of the GI-TS Data

Total scattering data were collected for the Al(111), Al(110), and Al(100) substrates. The theoretical critical angle α_*c*_ of aluminum oxide is about 0.02° at the X-ray energies used in these experiments. Therefore, the grazing incidence (GI) condition, facilitating surface sensitivity, is fulfilled by setting the incidence angle α between the incoming X-ray beam and substrate to 0.015 ± 0.001°. A second geometry, with an incident angle above α_*c*_ of around 0.05 ± 0.001°, was tested as well. Unlike the first geometry, this configuration leads to bulk penetration of the direct beam^52^ into the Al crystal. The conditions used for each experiment are summarized in Table 1. Moreover, to investigate the effect of first and second anodization of the substrates, the samples are labeled by their crystallographic direction and anodization stage, namely, Al(111) 1st, Al(111) 2nd, Al(110) 2nd, and Al(100) 1st.

**Table 1:** Condition Used for Each Experiment

experiment	condition
Al(111) 1st	below α_c_
Al(111) 2nd	above α_c_
Al(110) 2nd	below α_c_
Al(100) 1st	above α_c_

Each data set, regardless of the crystallographic orientation of the Al substrate, showed similar trends. At first, a series of sharp Bragg peaks are observed in the detector image (Figure 2a). As the anodization process progresses, these sharp peaks are gradually replaced by a series of broad rings (Figure 2b), which indicates the absence of a long-range order. The oxide layers, grown in this study, are thus amorphous, in line with previous studies.^41^

**Figure 2:**
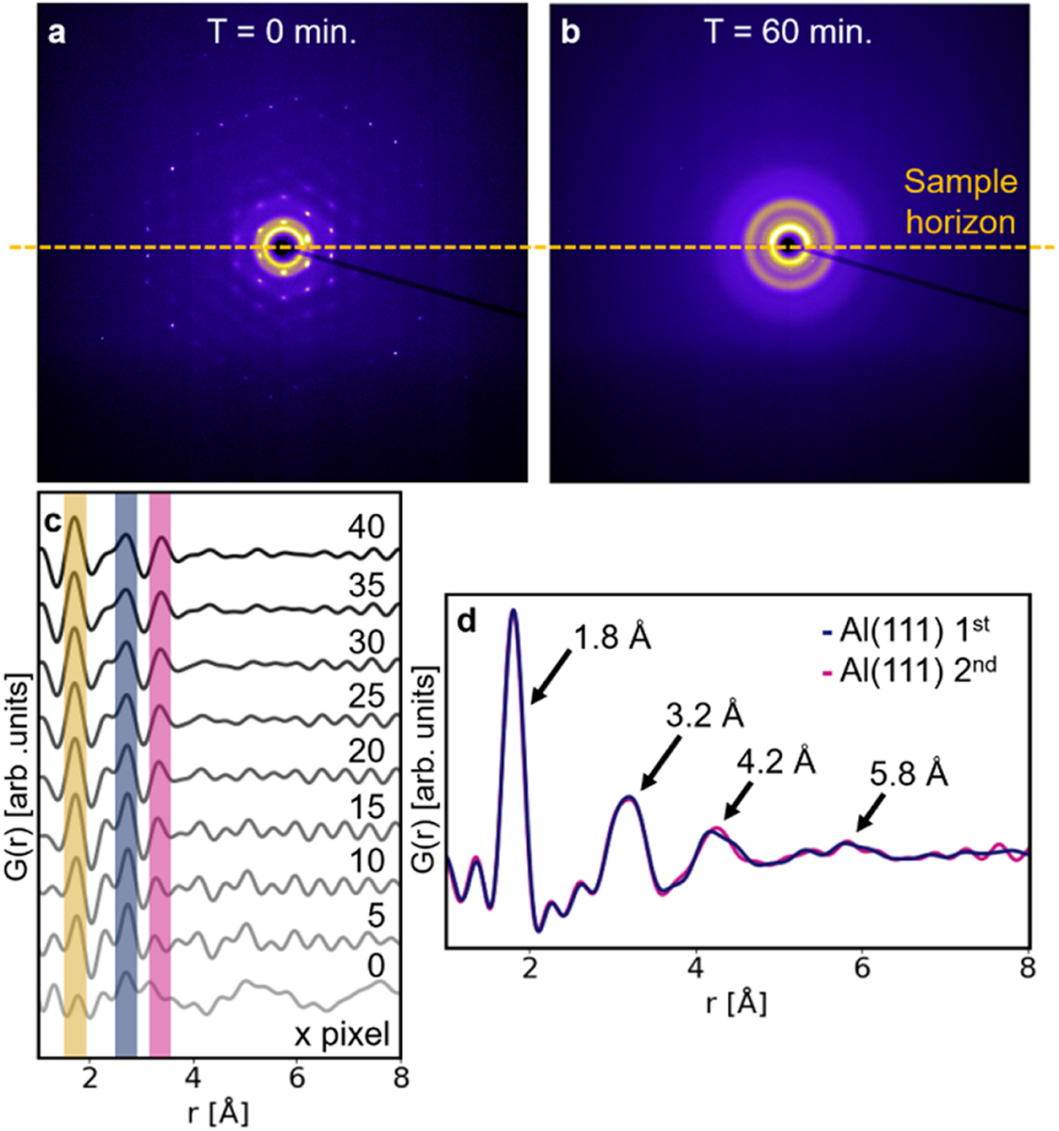
Detector images of the Al(111) 1st experiment at (a) *t* = 0 min and (b) *t* = 60 min (*t* refers to the time relative to the beginning of the experiment). (c) PDFs of the detector image shown in (a) obtained by masking the Bragg spots with different mask sizes, indicated in the form of the diameter of each circular mask, in number of pixels, next to each PDF (see Figure S2). (d) PDF of the end-product for the Al(111) 1st and 2nd experiments.

The presence of Bragg spots across the detector image, ranging from 4 spots for Al(111) 2nd to 45 spots for Al(111) 1st, makes it far from trivial to process data collected early in the reaction. Due to their *hkl* dependence, these Bragg spots are unevenly distributed across the detector image. Moreover, their relative intensity fluctuates from frame to frame, which results in statistical inaccuracy upon reduction of the data to 1D patterns. Keeping these spots in the integrated patterns does, however, result in the emergence of additional peaks and oscillations in the PDF that impede data analysis. Therefore, removing the contribution of the single-crystalline Bragg peaks from the integrated patterns is crucial for correct analysis of the data.

To address this issue, we employed the following method. First, the detector images were split into two parts for the signal scattering above and below the sample horizon, respectively. The region of raw detector images corresponding to the scattering below the sample horizon was omitted due to the poor signal-to-noise ratio caused by absorption in the sample and electrolyte (Figure 2a,b). Second, the initial 60 images of the data set were averaged for obtaining an improved signal-to-noise ratio. Based on this averaged data set, we have produced a map of Bragg spots positions and widths that were used to generate disk-shaped masks with Dioptas for further data treatment. Overexposed and unresponsive (“dead”) pixels were also masked and omitted from the data treatment.

In order to investigate the extent of the Bragg spots and their scattering, the size of the masking disks was gradually increased, starting from a radius of 5 pixels up to 40, i.e., a radius in millimeters ranging from 1 to 8 mm, with an increase in steps of 5 pixels (1 mm) (see Figure 2c). The PDF of the corresponding scattering patterns was obtained by setting the *Q*_max_ to 14.0 Å^–1^ to exclude residual high *hkl* index peaks. Even if these are nearly invisible in the raw detector images, they are amplified in the *F*(*Q*). The absence of remaining scattering signal from the Bragg peaks was thus investigated via the corresponding *F*(*Q*) of each data set, presented in Figure S3. The successful masking of the Bragg peaks is evidenced by their absence in the masked *F*(*Q*) data of all samples across various anodization times. In real space, the presence of the scattering from the Al substrate translates to the presence of a strong PDF peak at 2.73 Å corresponding to the first Al–Al pair in an Al face-centered cubic (FCC) structure indicated in blue in Figure 2c. For the 0 pixel-large masks, this peak is weak and large oscillations are observed instead throughout the PDF. This behavior arises from the presence of one or two intense Bragg peaks, whose Fourier transform gives rise to large oscillations in real space. As the Bragg peaks were more and more masked, this peak gradually decreased in amplitude. Instead, a set of new peaks appear. In the case of the Al(111) 1st data set, we can observe peaks at 1.7, 2.7, and 3.2 Å, shown in yellow, blue, and purple in Figure 2c, respectively. We can ascribe the resulting PDF to a native oxide present at the beginning of the Al(111) 1st experiment. Such protective native oxides, with thickness usually ranging from 2 to 5 nm^53^ commonly form on both pure and alloyed Al substrates. With our masking approach, we can conclude that the minimum diameter of disks necessary for proper masking of the Bragg peaks during PDF integration is 35 pixels or 7 mm.

In order to obtain a high-quality PDF of the oxide formed at the end of the anodization process, the last 60 detector images were averaged. Because of the absence of Bragg peaks on the detector images at this stage of the reaction, the Fourier transform of the scattering pattern was performed this time by setting the *Q*_min_ and *Q*_max_ to 0.7 and 17.5 Å^–1^, respectively. The PDFs of each experiment’s anodized product, shown in Figure 2d, were obtained with these range settings. A finite set of peaks, respectively, at 1.8, 3.2, 4.2, and 5.8 Å, characterize these PDFs. Beyond the peak at 5.8 Å, no distinct feature is observed. This indicates the absence of structural coherence in the resultant anodic oxide beyond this distance. We further noticed that the PDFs of all substrates are nearly identical, suggesting that a very structurally similar amorphous oxide grows on the substrate regardless of its crystallographic orientation. The obtained PDFs furthermore resemble those of the bulk amorphous oxide structure reported by Lamparter and Kniep.^41^ The last step in data processing consisted of obtaining PDFs of the complete time-resolved data sets. The total scattering patterns were averaged over 15 frames, yielding a time resolution of 15 s, which was found to be a good compromise between a reasonable signal-to-noise ratio and a sufficient time-resolution to observe structural changes. The data were thus integrated after the Bragg spots. The scattering contribution from the cell and electrolyte was subtracted from the experimental TS data, as presented in the previous section. To avoid any signal oversubtraction that may yield nonphysical features on the PDF, the scattering pattern of the cell containing only electrolyte was scaled down until it was always slightly less intense than the experimental sample TS pattern, around 98% of its intensity, avoiding signal undersubtraction as well, as presented in Figure S1. It is worth noting that during anodization, the NP-AAO/electrolyte interface is displaced and so are the X-rays scattered on the surface oxide. However, this displacement is about 10–100 times smaller than a single pixel of the used detector (200 × 200 μm^2^), meaning that such displacement is not perceived on the detector image and would not impact the resulting PDFs.

## 3 Results

### 3.1 Data Overview

For each experiment, measurements were initiated 60 s prior to applying a potential to the cell. Since the aim of the experiment was to observe the structure at all steps of the reaction, including the early stages, the *Q*_max_ was set down to 14.0 Å^–1^, as discussed in the previous section. We show the amplitude of the resulting PDFs as a function of time in the form of colormaps for the two Al(111) experiments (1st and 2nd steps) and Al(110) 2nd in Figures 3 and S4a, respectively. As mentioned in the methods, the data were recorded for 2.5 h, but Figures 3 and S4a show PDFs collected during the first 50 min, since no further change in the PDF is observed afterward. The full data sets are presented in Figure S5. Although the experiments yield very similar final structure (see Figure 2d), the pathway toward it differs from one sample to another, especially when comparing those probed during a first or second anodization. These differences are described in the sections below.

**Figure 3:**
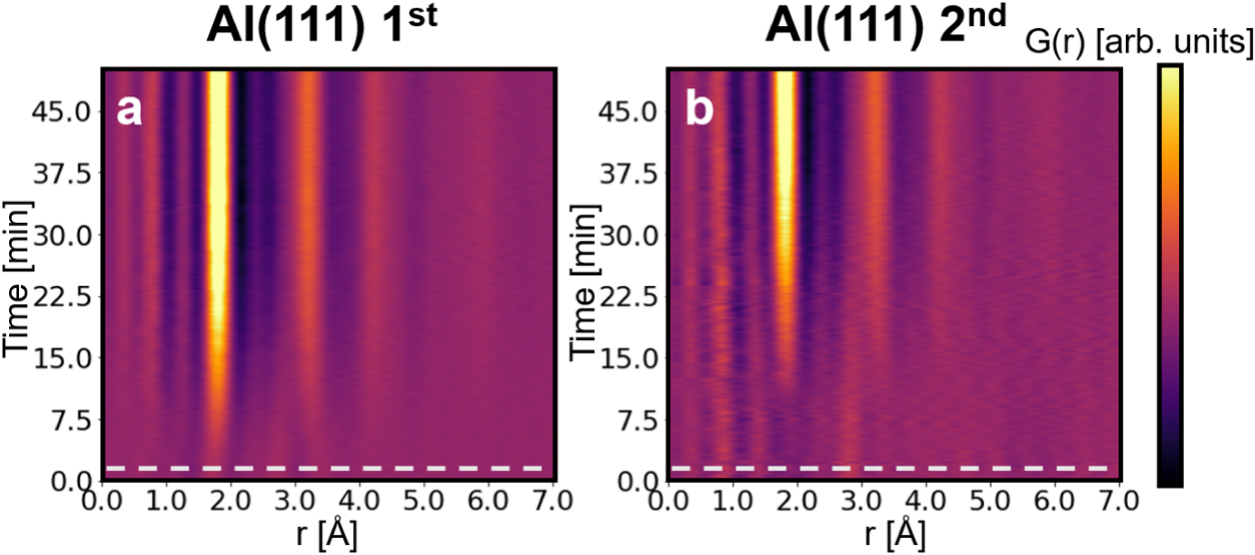
Color maps of the time-resolved PDF data sets obtained for experiments (a) Al(111) 1st and (b) Al(111) 2nd. The gray dotted line in both colormaps indicates the time at which the electric potential is applied in the cell.

In addition to the experiments discussed so far, an Al(100) surface was anodized and investigated at the ESRF under GI conditions (labeled Al(100) 1st). This time, *Q*_max_ was set to 13.0 Å^–1^. The corresponding PDFs contour plot is shown in Figure S6a. A zoom into the early stage of the reactions as well as the first peaks of the PDFs are shown in Figures 4, S4b, and S6b, for Al(111) 1st, Al(111) 2nd, Al(110) 2nd, and Al(100) 1st, respectively.

**Figure 4:**
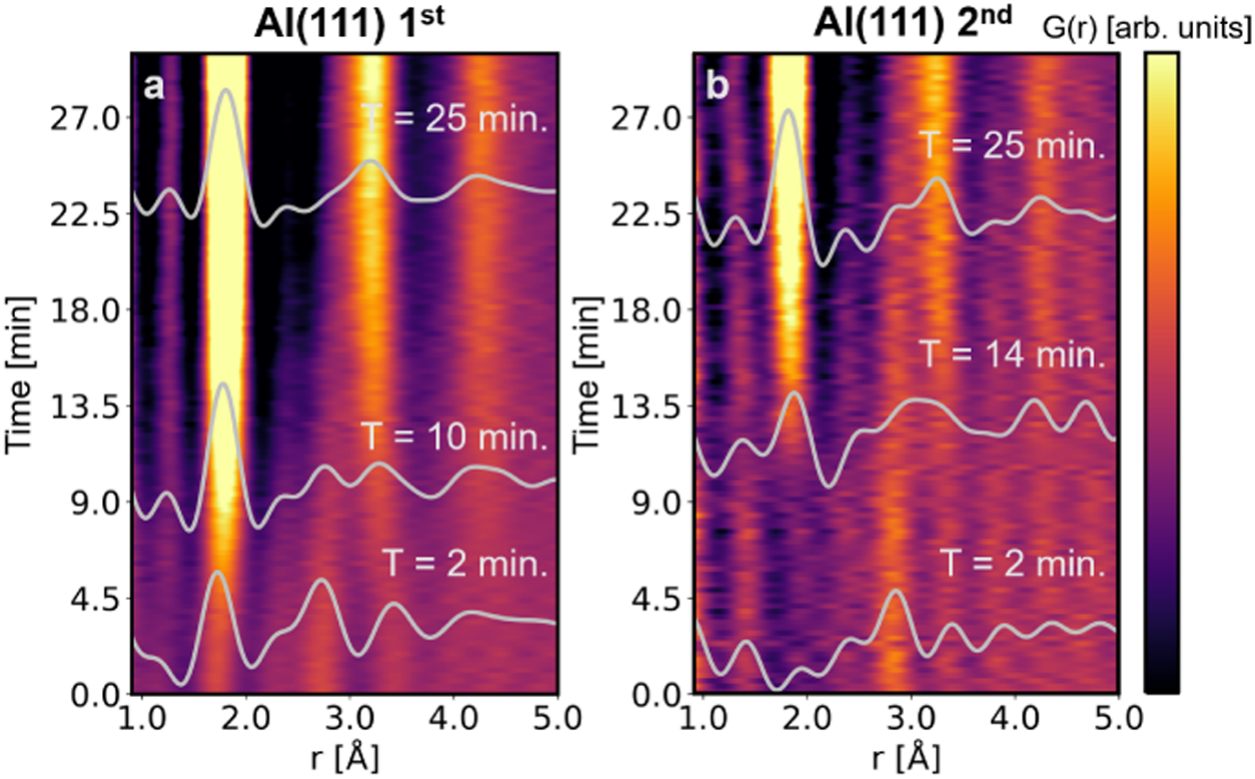
Colormaps and selection of PDFs representative of different steps in the anodization over time highlighted in gray for the experiments (a) Al(111) 1st and (b) Al(111) 2nd.

### 3.2 Structure Evolution of the NP-AAO during the First Anodization

At the beginning of the Al(111) 1st experiment (see Figures 3a and 4a), a set of peaks, at 1.7, 2.7, and 3.2 Å, are present in the PDF data, as highlighted at *t* = 2 min in Figure 4. After 10 min of anodization, these peaks shift in position, with peaks at 2.7 and 3.2 Å mentioned above even merging together. Moreover, a broad peak at 4.2 Å appears, revealing an extended ordering to a longer range at the surface. The PDF peak under 2.0 Å can be assigned to an aluminum–oxygen bond^54^ and indicates the presence of a native oxide at the surface of the Al(111) 1st from the beginning of the experiment. As mentioned earlier, the formation of native oxide with a thickness of a few nanometers at the surface of aluminum and aluminum alloys is to be expected.^53^ The PDF of the end-product (Figures 2d and 4a at *t* = 25 min) can be assigned to the structure of a formed NP-AAO layer, and its corresponding structure will be discussed later. Similar to Al(111) 1st, the Al(100) 1st data exhibit PDF peaks up to 5 Å at the beginning of the experiment (see Figure S6b). However, the first peak below 2.0 Å, assigned to the Al–O bond, is placed at a higher *r* value than in the Al(111) 1st experiment, namely, 1.9 Å instead of 1.7 Å. This difference will be discussed in the last section of this report. Throughout the anodization process, the PDF then evolves toward that of the end-product of the Al(111) 1st experiment.

### 3.3 Structure Evolution of the NP-AAO during the Second Anodization

For the second anodization step, illustrated by experiments Al(111) 2nd and Al(110) 2nd (Figures 4b and S4), no peak between 1.7 and 2.0 Å is observed at the beginning of the reaction, as evidenced by the PDF at *t* = 2 min, suggesting the absence, or absence of detection, of native oxide. Instead, only a peak at 2.85 Å, corresponding to the Al–Al distance in the Al FCC structure, is present.

The probed structure evolves upon anodization and gives rise to a very similar PDF at the end of the process, as observed after the first anodization (Section 4.2). In contrast to the former case, however, between *t* = 10 and 15 min, peaks below 2.0 and at 3.3 Å appear and shift their position until the final structure is reached, as highlighted at *t* = 14 min. An intermediate phase can thus be identified, involving the formation of an oxide, known as the barrier oxide. This intermediate barrier oxide structurally differs from the final NP-AAO.

Finally, for both experiments Al(111) 1st and Al(111) 2nd, a peak around 1.5 Å is observed at the early stage of the anodization process and weakens as the final structure is reached (Figure 4). This peak may be explained by the use of H_2_SO_4_ as the electrolyte: The expected S–O bond should give rise to a PDF peak around this position.^55^ However, a closer inspection in this r range indicates that the peak is located around 1.3 Å, which is too short to correspond to an S–O bond, especially in solution where this bond length is about 1.55 Å. Alternatively, it could correspond to O–O peroxo bridges that can be found in anodized aluminum oxides, as evidenced by theoretical studies.^56,57^ However, these studies specified that the peroxo bridges may only be stable in alkaline electrolytes, which contrasts with our experiments being carried out in acidic medium. Moreover, chromium­(III) and (VI) species, coming from the chemical etching of the sample surface prior to the second anodization step, are known to form on the Al surface as a result of the etching process.^58^ However, no such species are visible in the PDFs of experiment Al(111) 2nd, as we do not observe any peak at the expected positions of the Cr–O bond in Cr­(VI) and Cr­(III) species, around 1.6 or 1.9 Å, respectively.^59,60^ This could indicate that potential Cr species are so diluted at the sample surface that they fall below the detection limit of the applied TS scattering technique. Instead, this peak rather corresponds to a data treatment artifact originating from the limited Q-range in which the Fourier transform is performed.

## 4 Discussion

### 4.1 Atomic Structure of the NP-AAO Layer

The nearest-neighbor Al–O bond distances reportedly range between 1.65 and 2.0 Å depending on the number of O^2–^ oxyanions coordinated around one Al^3+^ cation.^54^

As presented in the previous section, all of the experiments, regardless of the crystallographic orientation of the substrate and preliminary etching of the surface, end up yielding oxide layers with almost identical PDFs (Figure 2d), consisting of a set of peaks below 7 Å. The absence of peaks beyond this value indicates the structure being amorphous. The peak at 1.8 Å matches the characteristic Al–O bond length between 1.65 and 2.0 Å. The second peak at around 3.2 Å is significantly broader and can be assigned to both Al–Al and O–O distances.

NP-AAO layers have been structurally investigated both via experiments and computational methods.^41,56,57,61^ Molecular dynamics (MD) simulations revealed that the NP-AAO structure consists of a network of undercoordinated [AlO_*x*_] polyhedra, typically [AlO_3_], [AlO_4_], and [AlO_5_] units. These polyhedra connect in a disordered manner via bridging oxygens and form ring structures. The size of these rings is characterized by the number of [AlO_*x*_] units contributing to it. Just like observed in our data, a previously reported experimental PDF of amorphous aluminum oxide^41^ shows peaks up to 7 Å, beyond which the PDF falls to zero. By combining both X-ray and neutron PDF with Reverse Monte Carlo (RMC) modeling, Lamparter and Kniep described the amorphous aluminum oxide network with the same building units as used in theoretical studies.^41^ Our experimental data show a very good agreement with the findings reported in their work. Therefore, our data indicate that an aluminum oxide network similar to the one studied by Lampartner and Kniep forms during the anodization process investigated here.

It should be noted that a chemical gradient exists within the pore structure between the inside and outside layer of the pore, with ions from the electrolyte more concentrated in the latter as adsorbed and absorbed species.^62^ This aspect impacts the growth process of the NP-AAO as well. However, due to the low electrolyte concentration (0.3 M) with respect to the dense aluminum oxide, their detection on the X-ray PDF is unlikely. Therefore, the impact of sulfate ions on growth will not be discussed further in this report.

### 4.2 Effect of the Two-Step Method on the Growth of NP-AAO

Two differences between the Al(111) 1st and Al(111) 2nd data sets can be pointed out. The first one relates to the Al–O bond length, while the second addresses the 2nd neighbor structure around Al atoms. First, changes in Al–O bond length during anodization occur in two distinct ways. To quantify these changes, the position of the first Al–O peak is tracked throughout the whole anodization process in each experiment. The peak maximum is extracted for each frame of the two data sets and then plotted against reaction time in Figure 5a. The tracked peak position varies within ±0.15 Å around 1.80 Å and demonstrates a difference in anodization pathways for 1st and 2nd anodization steps. Analogous analysis of the Al(110) 2nd and Al(100) 1st data is presented in Figure S7a,c.

**Figure 5:**
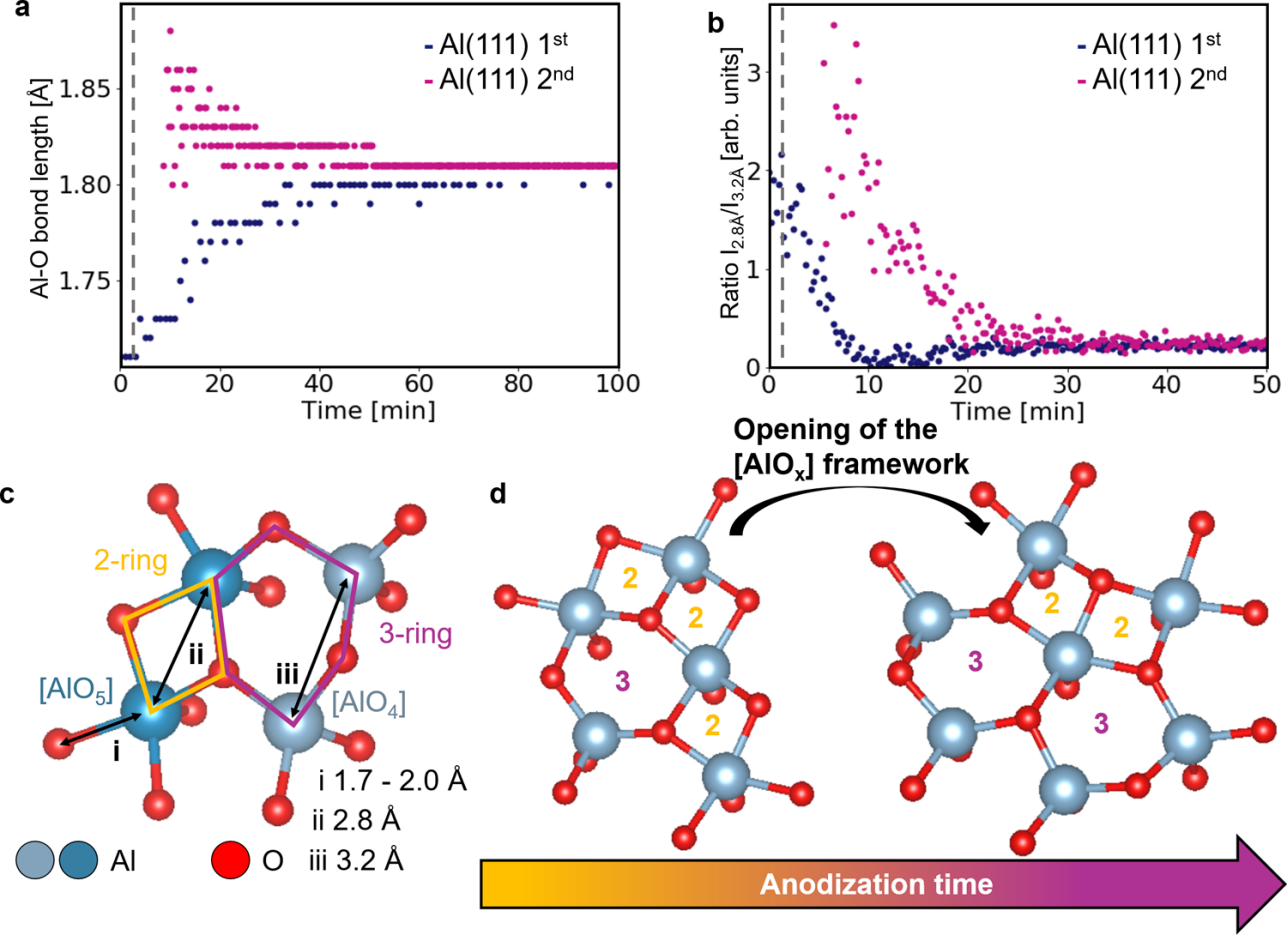
Evolution of (a) the Al–O bond length over time in the Al(111) 1st and 2nd experiments and of (b) the ratio of amplitudes of the first two Al–Al peaks at 2.8 and 3.2 Å over time in the Al(111) 1st and 2nd experiments. (c) Illustration of the different atomic pair distances observable in amorphous aluminum oxide. (d) Illustration of the structural reorganization process occurring in the amorphous aluminum oxide network throughout the studied anodization. In (a) and (b), the gray dotted line indicates the time at which the electric potential is applied in the cell.

At the beginning of the first anodization process of the Al(111) surface, i.e., in Al(111) 1st, the initial Al–O bond length is about 1.70 Å. This initial bond length is compatible with the one found in recent computational and experimental evidence of surface relaxation on crystalline aluminum oxide.^43^ In our experiment, the bond rapidly stretches and then dwells at 1.81 Å. This stabilization in the peak position indicates that the final oxide structure discussed above is reached. Meanwhile, in samples anodized for a second time after chemical etching of the grown NP-AAO and in the absence of detection of a native oxide, the initial Al–O bond length in the oxide formed after a few minutes of anodization is about 1.90 Å, which is longer than in the “bulk” oxide. As the barrier oxide forms in the final NP-AAO, the Al–O bond gradually shrinks to 1.81 Å. For the Al(110) 2nd experiment, the final bond length is 1.83 Å (Figure S7a), differing by about 1% from the Al(111) experiment’s final Al–O bond length. This small difference is likely due to calibration and instrumental variations rather than structural factors, as both data sets were processed with the same *Q*_min_ and *Q*_max_ while the sample position in the cell may slightly vary from one experiment to the other.

In the Al(100) 1st PDF data (Figure S6), a peak below 2.0 Å suggests the presence of a native oxide. Unlike Al(111), which initially showed an Al–O bond length of 1.70 Å, the native oxide in Al(100) has a bond length of around 1.9 Å (Figure S7c). This difference may be due to Al(100)’s lower atomic density, with only four first neighbors compared to six in Al(111). The lower atomic density allows for less steric hindrance between O atoms during anodization, resulting in a longer Al–O bond, which eventually decreases and stabilizes at 1.83 Å.

The second difference between the first and second anodization steps of the “two-step” method concerns the order of the 2nd neighbor distances. During the first anodization, two distinct peaks at 2.85 and 3.20 Å are observed on the PDF data. Meanwhile, during the second anodization, a broad peak covers the same pair distance range. This broad peak corresponds to a broad distribution of distances and gradually shifts from around 2.75 Å toward around 3.20 Å, which is the distance expected for the “bulk” NP-AAO (Figure 2d). These peaks can be assigned to Al–Al distances, either in Al FCC (2.86 Å in the bulk structure), at the beginning of the experiment, or in an oxide phase, when the aluminum oxide layer starts to be observed. In the latter case, a short Al–Al distance, typically below 3.0 Å, may arise from two edge-sharing [AlO_*x*_] polyhedra, as indicated by (ii) in Figure 5c. Conversely, an Al–Al distance above 3.0 Å can be assigned to highly coordinated, corner-sharing [AlO_*x*_] polyhedra, typically *x* ≥ 4. These corner-sharing polyhedra give rise to an Al–Al pair distance indicated by (iii) in Figure 5c. They, furthermore, give rise to a more open structure with lower density than edge-sharing polyhedra would. Note that this analysis provides a qualitative estimate of the ring structure within the oxide film. Given its amorphous structure, a distribution of larger ring sizes can be expected, although it is not accounted for in this analysis. Thorough studies employing, e.g., RMC modeling of the PDF data at different times may allow us to retrieve quantitative information on the structural reordering at play during the anodization process. However, this kind of analysis is beyond the scope of this work.

The presented differences between samples undergoing a first or second anodization can be interpreted as follows. In the case of the first anodization, the surface bears, from the beginning, a native oxide exhibiting some order at the local scale, which translates to a dense disordered network of [AlO_*x*_] polyhedra connected through both corners and edges. In the case of the second anodization, a more disordered oxide barrier is observed. After the electric potential is applied in the case of the first and the second anodization, the oxide barrier formation ends after 11 s and after 8–18 s, respectively (as estimated from the electrochemical current density data in Figure S8). The structure of such a network has previously been determined in MD studies, where the oxide layer atomic structure mainly consisted of ring structures composed of two to three [AlO_*x*_] polyhedra. Similarly, an experimental scanning tunneling microscopy study on surface native oxide of NiAl (110) alloys revealed local order comprising both edge- and corner-sharing [AlO_4_] and [AlO_5_] polyhedra.^63^ More recently, the investigation of the reconstructed Al_2_O_3_(0001) surface via atomic force microscopy revealed a similar network of undercoordinated [AlO_*x*_] polyhedra sharing edges and corners.^43^ The radial distribution functions extracted from their structural model match well with our experimental PDFs, where the average Al–O bond length locates around 1.8 Å and Al–Al between 2.8 and 3.2 Å.

The different types of ring structures proposed in the literature give rise to different PDF peaks (shown in blue and purple in Figure 2c), whose relative amplitude varies throughout the anodization. The ratio of their amplitudes is plotted as a function of time in Figure 5b for Al(111) ions 1st and 2nd. Figure S7b,d show equivalent analyses for Al(110) 2nd and Al(100) 1st, respectively. For all experiments, the calculated ratio is above 1 at the beginning of the anodization process, meaning that the peak at 2.8 Å is more intense than the one at 3.2 Å. Throughout the anodization, the corner-sharing Al–Al peak at 3.2 Å gains amplitude until the ratio dwells around 0.2. Ultimately, this can be interpreted as a solid-state rearrangement, where rings made up of two [AlO_*x*_] polyhedra open to form even larger ring-like structures consisting of three or more polyhedra as illustrated in Figure 5d. For the sample investigated during the second anodization, the barrier oxide that forms during anodization shows only a broad distribution of pair distances between 2.7 and 3.2 Å. This indicates a broader distribution of ring sizes in the initial oxide structure which subsequently reorganize to reach the NP-AAO structure previously discussed.

The different formation behaviors presented in this section may be related to the formation mechanism of the porous aluminum oxide network. Previous experimental evidence suggested that the growth of NP-AAO might be governed by the combined dissolution of oxide at the oxide/electrolyte interface and formation of oxide at the metal/oxide interface.^6^ The increase in Al–O bond length in the Al(111) 1st sample suggests that the native oxide undergoes mechanical stress that is released as the “bulk” oxide grows.^64,65^ On the other hand, the oxide layer structure in the Al(111) 2nd and Al(110) 2nd samples exhibits a longer Al–O bond at the step where the barrier oxide forms than in the final NP-AAO structure, and shrinks as it grows during anodization. In this case, formation of oxide on Al freshly exposed to electrolyte means that the Al–O bond would not be constrained by mechanical stress as for the first anodization samples.^66,67^ Moreover, the presence of a higher amount of small [AlO_*x*_] rings at the beginning of the reaction is not in agreement with the distribution of ring sizes estimated by MD. It can therefore be understood as an out-of-equilibrium system that tends toward the more stable, more opened network structure of “bulk” amorphous aluminum oxide.^56,57^ These elements are in line with the stress growth model of NP-AAO, which suggests that the growth is driven by the presence of mechanical stress in the network. The amorphous nature of the NP-AAO atomic structure allows the strains to be relaxed via solid-state rearrangement.

### 4.3 A Critical Assessment of the Surface Sensitivity of These Experiments

Our data collected below the critical angle closely resembles that collected above it. Two possible interpretations can be drawn from that. One is that the topmost oxide structure is structurally similar to the one in the “bulk” NP-AAO, which grows across tens of nanometers. Alternatively, we may have probed the bulk in both geometries instead of selectively probing the surface when working below the critical angle. However, the clear observation of a thin, few nm-thick native oxide in experiments Al(111) 1st and Al(100) 1st that were measured below and above the critical angle, respectively, suggests that in both cases the surface of the sample is selectively probed. Conversely, the two experiments done during the second anodization step, Al(111) 2nd and Al(110) 2nd, were performed above and below the critical angle, respectively, and displayed very similar behavior. They both started from a thin oxide layer (undetected on the PDF data) and later formed a highly disordered barrier oxide layer with an Al–O bond close to 2.0 Å that shrinks toward 1.8 Å as the barrier oxide layer grows into the NP-AAO. Investigating a porous amorphous structure can make it difficult to set the angle between the incident X-ray beam and the surface below the actual critical angle. Both the chemical composition and structure of the surface impact it, but cannot be determined exactly for an amorphous aluminum oxide ranging around Al_2_O_3_ in chemical composition.^63^ Moreover, the fulfillment of the GI conditions relies on a low roughness of the surface, which is not the case from the moment the porous oxide forms onward.^53,68,69^ However, the fact that we do observe distinct initial states in the different PDF data sets indicates that the experiments were in fact surface sensitive enough to reveal surface structure. In the case of the second anodization step, the substrates’ roughness is high due to the etching process prior to the experiment. This procedure removes any aluminum oxide from the surface, as typical for the “two-step” anodization method,^32^ and comes with a roughening of the surface, since the selective removal of nanoporous alumina leaves the Al substrate patterned with nanoconcaves. Therefore, this surface roughness may prevent the fulfillment of the GI conditions and thus hinder the observation of the scattering signal from a potential native oxide at the beginning of the experiment. Without the GI condition fulfilled, some bulk penetration of the X-ray beam may occur, and detection of the Al substrate may happen. In other words, the surface sensitivity of the technique may be lost. The theoretical bulk penetration can thus be calculated geometrically from the used incidence angles (0.015 and 0.05° in the two geometries applied here, respectively) and the width of the single crystal (6 mm) (see eq 1 below, where d (in mm) is the penetration depth, α the incident angle (in deg), and *w* (in mm) the width of the Al single crystal), and yielding a theoretical penetration depth of about 1.6 and 5.2 μm, respectively. This value is in the same order of magnitude as the vertical beam size (2 and 5 μm at DESY and ESRF, respectively), which means that this width is not substantially affecting the theoretical penetration depth of the X-ray beam. Given that at the end of the anodization reactions we do not observe any Bragg peak from the Al substrate anymore, we can conclude that the film thickness should be thicker than the theoretical penetration depth. In fact, the final film thicknesses calculated for each sample based on the current density data and parameters presented in Table S1, shown in Table S2, are all thicker than the theoretical penetration depth. Moreover, scanning electron microscopy (SEM) micrographs of Al surfaces anodized in the same conditions as reported in this report are shown in Figure S9 and exhibit oxide thicknesses of the same order of magnitude. In addition to the surface roughness, the limited thickness of the native oxide film and its amorphous nature make it a poor X-ray scatterer and its corresponding signal may fall below the detection limit.

1 d=w·sinα

Therefore, performing experiments with an incidence angle within the same order of magnitude as the theoretical critical angle, whether it is above or below, allows us to perform TS measurements on real surfaces that present some roughness and virtually allows us to achieve surface sensitivity. The key step then is to find the right strategy for data processing in order to retrieve the scattering signal from the surface. The fact that the background signal from the substrate is concentrated in the Bragg spots helps in the sense that the regions where they overlay with the film signal are masked out, leaving the majority of the azimuthal range as the direct film signal plus the isotropic background from the cell. At the applied high-energy photons, small rotational adjustments of the cell around the film normal in the order of 10^–2^ ° sufficed to orient the substrate out of the Bragg condition and overall minimize their intensity and leave more of the dynamic range of the detector for the film signal. This strategy enabled reaching the extraordinary surface sensitivity down to the native oxide of only a few nanometers thickness in such a complex sample environment.

## 5 Conclusions

In this contribution, we demonstrated the feasibility of time-resolved X-ray total scattering experiments performed in grazing incidence conditions with samples under realistic conditions. The PDFs of the amorphous aluminum oxide growing on Al crystals could be extracted in a time-resolved manner, despite the strong scattering contributions from the single crystals and the sample environment, i.e., the cell walls and electrolyte. The anodization process was carried out on aluminum single crystals with different crystallographic orientations, namely, (111), (110), and (100). The analysis of the PDF of the anodized amorphous oxide at the end of each reaction revealed a similar structural motif within the amorphous oxide regardless of the substrate’s crystallographic orientation. However, their formation pathways differ. It was influenced not only by whether the aluminum substrate was anodized for the first or second time but also by the removal of oxide with chromic acid, intrinsic to the preparation of the second anodization step. In addition to the first anodization itself, this stripping of oxide likely contributes to the observed differences in oxide formation. We found that, during the anodization, the aluminum oxide network expands whether or not the surface is treated with chromic acid. When the surface is stripped of the porous oxide, as prior to the 2^nd^ anodization step in the “two-step” anodization method, amorphous oxide forms via a more disordered barrier oxide. Our results support the mechanical stress-driven growth of NP-AAO on aluminum substrates, where changes in the bond lengths are evidence for mechanical strains at play during the studied anodization process.

## Supplementary Material



## Notes

Data processing; pair distribution function data and analysis; effect of crystallography facet on the evolution of the Al–O bond length; electrochemical current density; and SEM micrographs (PDF)

## ○

Leiden Institute of Chemistry, Leiden University, Einsteinweg 55, 2333 CC Leiden, The Netherlands
