# Supplementary material for: In Situ Grazing Incidence X‑ray Total Scattering Reveals the Effect of the “Two-Step” Method for the Anodization of Aluminum Surfaces
Source: ACS Appl Mater Interfaces. 2025 Aug 11;17(33):46887–98. doi: 10.1021/acsami.5c05251 (PMC12371698; doi:10.1021/acsami.5c05251)
Supplement: Supplementary file 1 [file am5c05251_si_001.pdf]

Supporting Information

In situ grazing incidence X-ray total scattering reveals the effect of the "two-step" method for the anodization of aluminum surfaces

Nicolas P. L. Magnard*[1,2], Giuseppe Abbondanza[3], Laura S. Junkers[1], Lorena Glatthaar[4], Andrea Grespi[5,6], Alexander Spriewald Luciano[4], Fernando Igoa Saldaña[7], Ann-Christin Dippel[7], Nikolay Vinogradov[8], Herbert Over[4], Kirsten M. Ø. Jensen*[1], and Edvin Lundgren*[5]

[1]Department of Chemistry, University of Copenhagen, DK-2100 Copenhagen, Denmark
[2]Current affiliation: Leiden Institute of Chemistry, Leiden University, Einsteinweg 55, 2333 CC Leiden, The Netherlands
[3]Department of Physics, Chalmers University of Technology, Chalmersplatsen 4, Gothenburg 41296, Sweden
[4]Institute of Physical Chemistry, Justus Liebig University, Heinrich-Buff-Ring 17, D-35392 Giessen, Germany
[5]Division of Synchrotron Radiation Research, Lund University, Professorsgatan 1, 22363 Lund, Sweden
[6]NanoLund, Lund University, Professorsgatan 1, 22363 Lund, Sweden
[7]Deutsches Elektronen-Synchrotron DESY, Notkestraße 85, 22607 Hamburg, Germany
[8]MAX IV Laboratory, Lund University, Fotongatan 2, 22484 Lund, Sweden

July 14, 2025

Corresponding authors' email: n.p.l.magnard@lic.leidenuniv.nl, kirsten@chem.ku.dk, edvin.lundgren@sljus.lu.se

1 Data reduction process

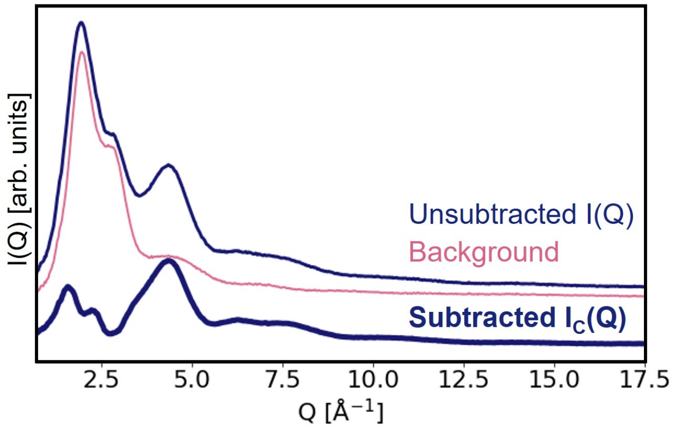

Figure S1: Example of background subtraction of the TS data. The presented TS data correspond to the end-product of the anodization of an Al(111) surface. The experimental TS data are presented in dark blue, the background, consisting in the scattering pattern of a PEEK cell filled with sulfuric acid, is shown in magenta, and the subtracted data in dark blue as a bold line.

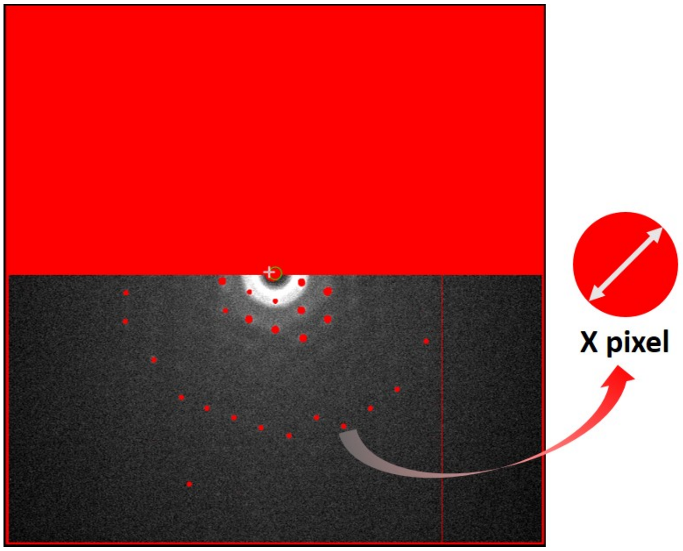

Figure S2: Example of mask used to process the detector images. The disk-shape mask used for the Bragg spots has its diameter X varied to study the extent of the scattering signal from it, as presented in Fig. 2c. The mask is represented in red and the detector image in a grayscale.

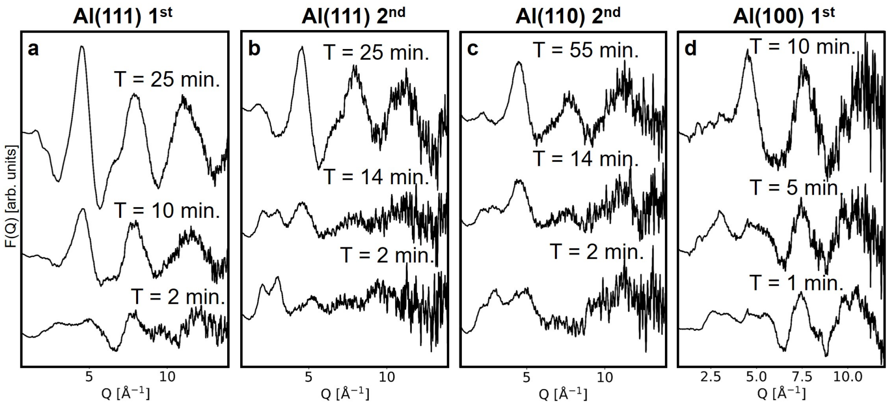

Figure S3: Scattering functions F(Q) representative of the different steps of anodization during the experiments a) Al(111) 1st, b) Al(111) 2nd, c) All(110) 2nd and d) Al(100) 1st.

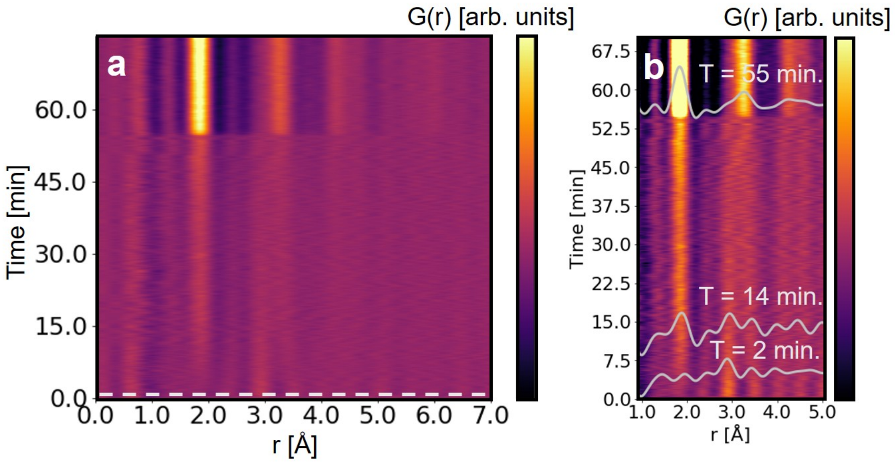

Figure S4: a) Colormap of the time-resolved PDF data collected during the Al(110) 2nd experiment. The grey dotted line indicates the time at which the electric potential is applied in the cell. b) Zoom into the first three PDF peaks of a) with PDFs representative of the different anodization stages highlighted in gray. Note: It can be noticed that a step appears around t = 52 minutes. This is due to a change in the attenuation of the beam, used at the beginning of the experiments to protect the detector from the intense Bragg peaks. The attenuator was removed during the experiment when the Bragg peaks disappeared.

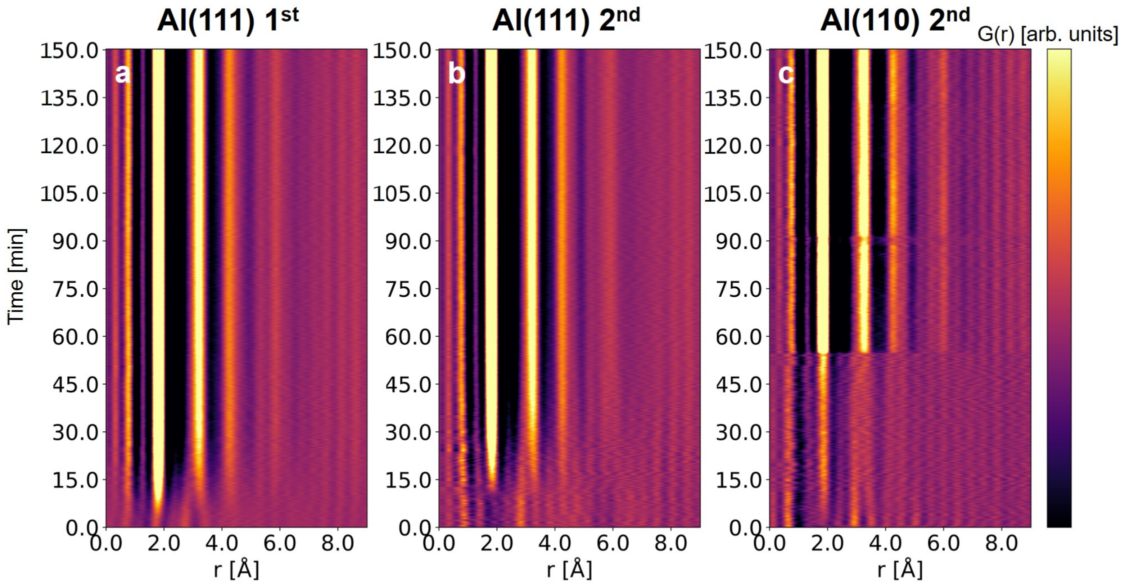

Figure S5: Colormaps of the time-resolved PDF data collected during a) the Al(111) 1st experiment, b) the Al(111) 2nd experiment and c) the Al(110) 2nd experiment throughout the whole time of recording, i.e., 2.5 hours.

2 Time-resolved X-ray TS experiments on the Al(100) surface at ESRF

Time-resolved X-ray TS experiments were carried out during the anodization of a Al(100) surface at ESRF. The TS data were corrected using the integrated scattering signal from the PEEK cell filled with sulfuric acid measured at DESY. The Fourier transform to obtain the PDFs was performed in a narrower Q-range, namely with a Q_{min} and Q_{max} to 0.7 and 13.0 $Å^{-1}$. For these reasons, a direct comparison of these data to the ones presented in the main manuscript cannot be carried out. However, the observation of similar trends in the evolution of the PDF over the anodization process reveals that a similar process is occurring on the Al(100) surface as on Al(111) and (110) surfaces.

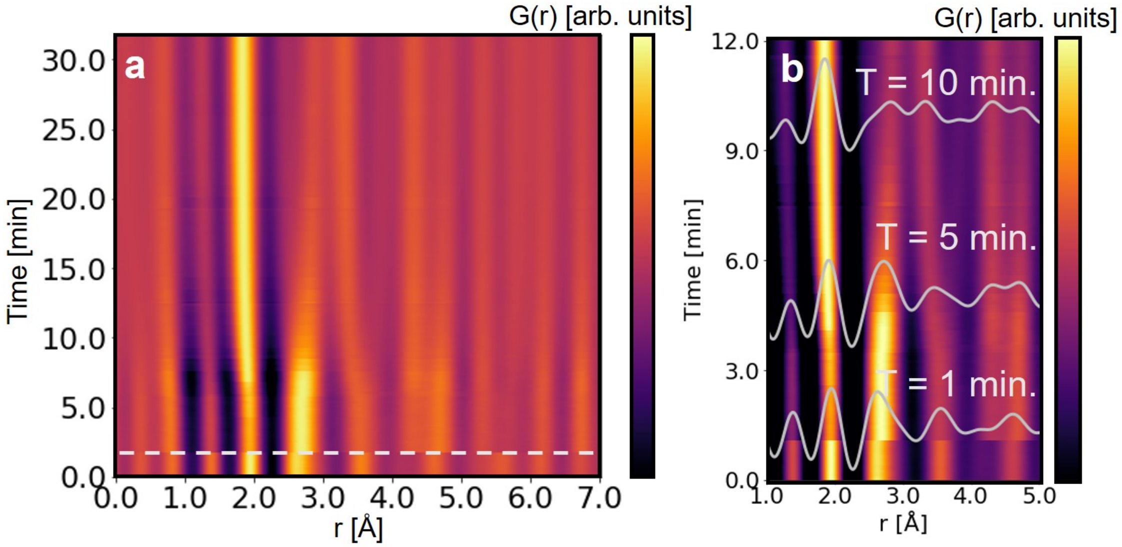

Figure S6: a) Colormap of the time-resolved PDF data collected during the Al(100) 1st experiment. The grey dotted line indicates the time at which the electric potential is applied in the cell. b) Zoom into the first three PDF peaks of a) with PDFs representative of the different anodization stages highlighted in grey.

3 Effect of crystallographic facet on the Al-O bond length evolution

In experiments where the Al(110) substrate was used, the Al-O bond length closely resembles those of the ”bulk oxide” throughout the anodization process, from the formation of the barrier oxide to its growth, remaining on average around 1.82 +/- 0.01 Å (see Fig. SS7). On the other hand, on the etched Al(111) substrate a larger shift in the bond length of about 0.15 Å is observed. (Fig. 5a) This difference in bond length change can be related to the packing density of each crystallographic facet. The Al(111) facet is densely packed with each surface Al atom having 6 in-plane nearest neighbors at 2.86 Å, while the Al(110) facet has only 2, and 2 neighbors at 4.05 Å. In the Al(110) case, the O atoms have thereby more room to adsorb at the surface and form longer chemical bonds to Al atoms, yielding a less dense barrier oxide. As the anodization process carries on and more oxygen is incorporated into the oxide layer, the network densifies and the average Al-O bond length shrinks. Since the Al(111) surface is already densely packed, the shrinkage in the Al-O bond length happens to a lesser extent.

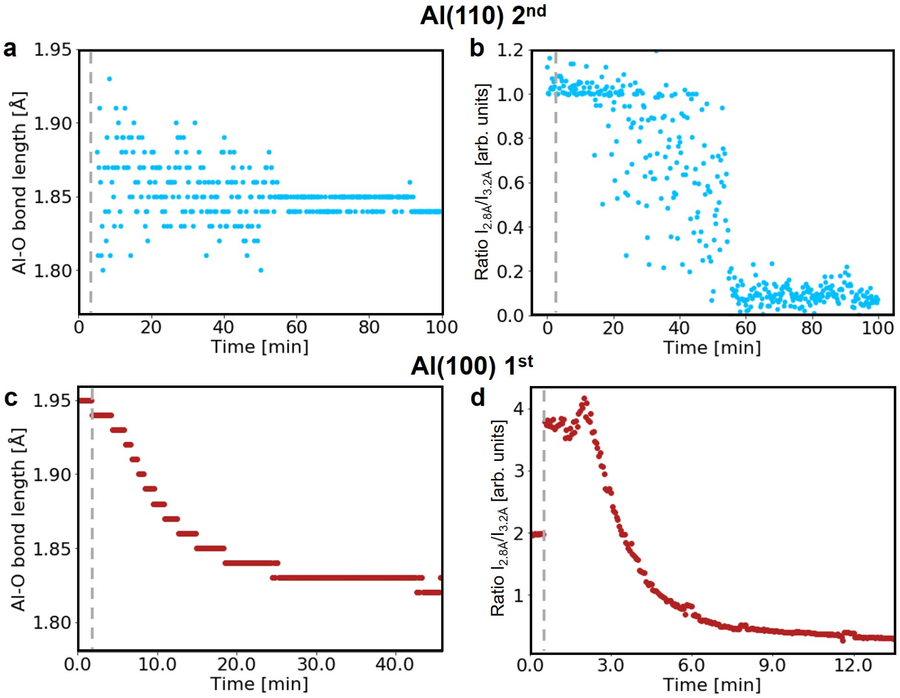

Figure S7: a) Evolution of the Al-O bond length over time in the Al(110) 2nd experiment. b) Evolution of the ratio of the amplitudes of the first two Al-Al peaks (at 2.8 and 3.2 Å respectively) over anodization time, for the same experiment. c) Evolution of the Al-O bond length over time in the Al(100) 1st experiment. d) Evolution of the ratio of the amplitudes of the first two Al-Al peaks (at 2.8 and 3.2 Å respectively) over anodization time, for the same experiment. The grey dotted lines indicate the time at which the electric potential is applied in the cell.

4 Electrochemical current density

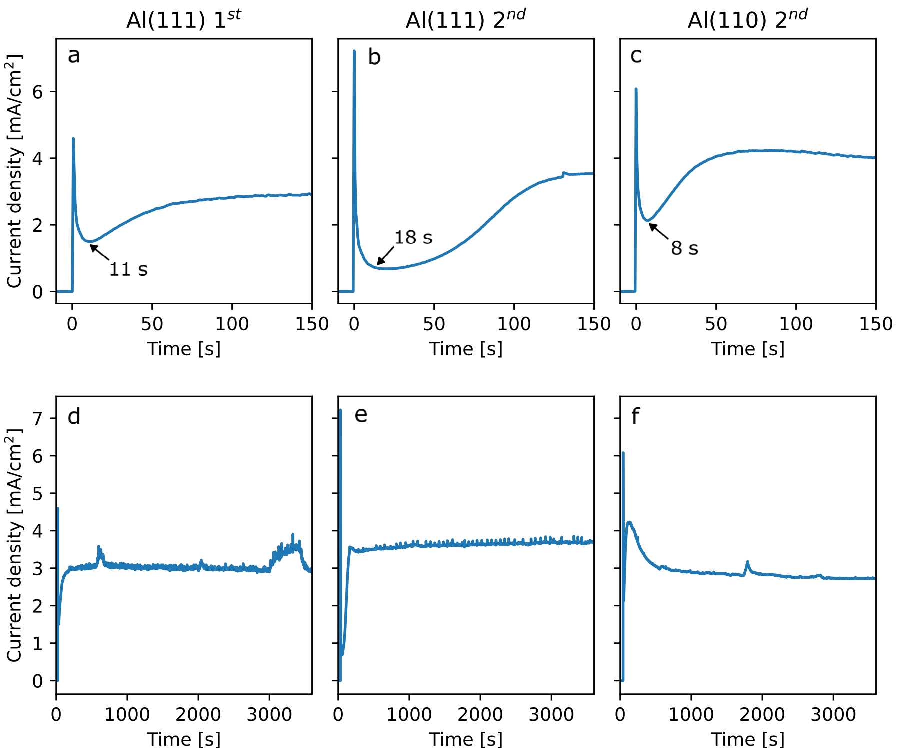

Figure S8: Current density profiles measured during anodization at 25 V in 0.3 M H_2SO_4. Panels (a–c) show the current density over the first 150 s for: (a) Al(111) – first anodization, (b) Al(111) – second anodization, and (c) Al(110) – second anodization. Panels (d–f) display the corresponding current density profiles for the same experiments shown in (a–c), respectively.

Fig. S8 (a–c) displays the electrochemical current density measured during the initial stages of anodization of aluminum in 0.3 M H_2SO_4 at 25 V. A distinct local minimum is observed in each curve, corresponding to the transition from compact barrier oxide growth to the formation of nanoporous alumina. This feature is characteristic of the onset of pore nucleation, where the initially insulating barrier layer becomes unstable and gives way to field-assisted dissolution and pore formation.

Fig. S8 (d–f) shows the full current density profiles for the same experiments, providing insight into the steady-state anodization behavior and long-term stability of the process.

The current density data were also used to calculate the total thickness of the nanoporous alumina layers formed, by applying Faraday's law under the assumption of 100% current efficiency for alumina formation:

$$d_{oxide} = PBR \cdot \frac{M_{Al}}{nF\rho_{Al}} \cdot J \cdot t, \quad (1)$$

where PBR is the Pilling-Bedworth ratio, M_{Al} and ρ_{Al} are the molar mass and the density of Al, respecitvely, n is the number of electrons involved in the oxide formation, F is the Faraday constant, J is the current density and t is the time elapsed. The constants used in this equation are shown in Table S1. From the total thickness and the anodization time, the average oxide growth rate was determined for each experiment. These values are summarized in Table S2.

**Name**	**Value**
PBR	1.62
M_{Al}	27 g/mol
ρ_{Al}	2.7 g/cm^3
n	6
F	96485 C/mol

Table S1: Values used to estimate the oxide thickness using Faraday's equation. The PBR value was found in literature for NP-AAO prepared in the same electrolyte and anodizing potential used in this work (i.e., H_2SO_4 and 25 V.)[1]

**Sample**	**Total Thickness (µm)**	**Average Growth Rate (nm/s)**
Al(111) – 1st anodization	12.5	1.39
Al(111) – 2nd anodization	15.4	1.71
Al(110) – 2nd anodization	10.0	1.37

Table S2: Total NP-AAO thickness and average growth rate calculated from electrochemical current density using Faraday's law.

5 Electron microscopy

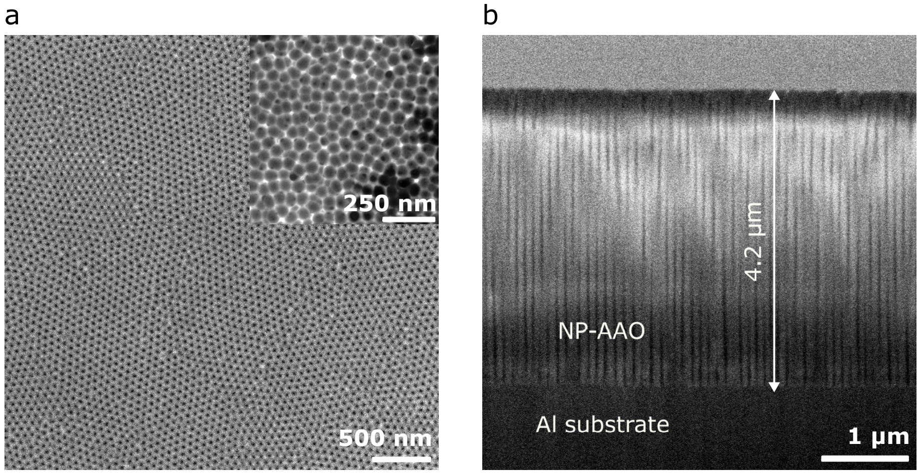

Figure S9: (a) Top-view SEM micrograph of NP-AAO fabricated by anodizing polycrystalline Al in 0.3 M H_2SO_4 at 25 V and 14 °C for 1 h. The inset shows the underlying nano-concave pattern revealed after oxide removal using a chromic/phosphoric acid mixture. (b) Cross-sectional FIB-SEM micrograph NP-AAO, displaying the interface between the porous oxide and the Al substrate. The NP-AAO thickness was measured from the micrograph and corrected by $\sin(\alpha)$, with $\alpha = 52°$ corresponding to the stage tilt. Based on the observed thickness of 4.2 μm, we could estimate a growth rate of 1.17 nm/s. This is in agreement with what estimated by means of current density analysis in Table S2. Note that although the crystallographic nature of the substrates used on these samples is different from those used in the manuscript, previous studies[2,3] as well as results from this manuscript, indicate that the final structure of the NP-AAO is the same.

References

(1) Arurault, L. *Transactions of the IMF* **2008**, *86*, 51–54.

(2) Vinogradov, N. A.; Harlow, G. S.; Carlà, F.; Evertsson, J.; Rullik, L.; Linpé, W.; Felici, R.; Lundgren, E. *ACS Applied Nano Materials* **2018**, *1*, 1265–1271.

(3) Evertsson, J.; Vinogradov, N. A.; Harlow, G. S.; Carlà, F.; McKibbin, S. R.; Rullik, L.; Linpé, W.; Felici, R.; Lundgren, E. *RSC advances* **2018**, *8*, 18980–18991.
